# Effectiveness and cost-effectiveness of an electronic mindfulness-based intervention to improve maternal mental health in the peripartum: study protocol for a randomised controlled trial

**DOI:** 10.1186/s13063-023-07746-7

**Published:** 2023-11-23

**Authors:** Grace Branjerdporn, Kerri Gillespie, Elizabeth Martin, Vivianne Kissane, Alex De Young, Tatjana Ewais, Kathleen Goldsmith, Susan Wilson, Sam Adhikary, Greg McGahan, Constanze Schulz, Michael Beckmann

**Affiliations:** 1 Mater Health, Annerley Road Campus, South Brisbane, QLD 4101 Australia; 2 Mater Research Institute – University of Queensland, Whitty Building (Ground Floor), Annerley Road, Mater Hill, South Brisbane, QLD 4101 Australia; 3 Wesley Research Institute, 451 Coronation Drive, Auchenflower, Qld 4066 Australia; 4 Peach Tree Perinatal Wellness Inc, 293 Ellison Rd, Geebung, QLD 4034 Australia; 5 Queensland Centre for Perinatal and Infant Mental Health, Children’s Health Queensland - Queensland Health, Nundah, QLD 4012 Australia; 6 School of Medicine and Dentistry, Griffith University, Gold Coast Campus, Parklands Drive, Southport, QLD 4222 Australia; 7 Faculty of Medicine, University of Queensland, 288 Herston Rd, Herston, Qld 4006 Australia; 8 Children’s Health Queensland, Queensland Health, 501 Stanley St, South Brisbane, QLD 4101 Australia

**Keywords:** Mindfulness, Maternal mental health, Maternity, Peripartum, Intervention

## Abstract

**Background:**

Perinatal women are highly vulnerable to developing mental health issues and particularly susceptible to a recurrence of psychiatric illness. Poor mental health during the perinatal period can have long-term impacts on the physical and psychiatric health of both mother and child. A potentially useful strategy to improve women’s mental health is through a mobile application teaching mindfulness, an evidence-based technique helping individuals focus on the present moment.

**Methods:**

A mixed method, prospective randomised controlled trial. The study group comprise women aged 18 years and over, who are attending the public and private maternity clinics at Mater Mothers’ Hospital. A sample of 360 prenatal women will be randomised into the intervention group (with the use of the mindfulness app) or usual care. Participants will remain in the study for 11 months and will be assessed at four timepoints for changes in postnatal depression, mother-infant bonding, and quality of life. A cost-effectiveness evaluation will also be conducted using quality-adjusted life year (QALY) calculations. A random selection of intervention participants will be invited to attend focus groups to give feedback on the mindfulness app.

**Discussion:**

Previous studies have found mindfulness interventions can reduce stress, anxiety, depression, and sleep disturbances in a prenatal population. The risks of the intervention are low, but could be of significant benefit for women who are unable to attend face-to-face appointments due to geographical, financial, or time barriers; during endemic or pandemic scenarios; or due to health or mobility issues.

**Trial registration:**

This study was approved by the Mater Misericordiae Human Research Ethics Committee (83,589). Australian New Zealand Clinical Trials Registry (ANZCTR) ACTRN12622001581752 (https://www.anzctr.org.au/Trial/Registration/TrialReview.aspx?id=385107&isReview=true). Registered on 22 Dec. 2022.

**Supplementary Information:**

The online version contains supplementary material available at 10.1186/s13063-023-07746-7.

## Administrative information

Note: the numbers in curly brackets in this protocol refer to SPIRIT checklist item numbers. The order of the items has been modified to group similar items (see http://www.equator-network.org/reporting-guidelines/spirit-2013-statement-defining-standard-protocol-items-for-clinical-trials/).

**Table:** 

Title {1}	Effectiveness and cost-effectiveness of an electronic mindfulness-based intervention to improve maternal mental health in the peripartum: A randomised controlled trial
Trial registration {2a and 2b}	Australian New Zealand Clinical Trials Registry (ANZCTR) Trial ID: ACTRN12622001581752
Protocol version {3}	Protocol version 3.1, Dated 05/09/2022
Funding {4}	Betty McGrath Seeding Grant—Mater Foundation.
Author details {5a}	Dr Grace Branjerdporn, Mater Health Ms Kerri Gillespie, Mater Research Institute - University of Queensland Dr Elizabeth Martin, Wesley Research Institute Ms Vivianne Kissane, Peach Tree Dr Alex De Young, Queensland Centre for Perinatal and Infant Mental Health Dr Tatjana Ewais, Mater Health Ms Kathleen Goldsmith, Mater Health Dr Susan Wilson, Children’s Health Queensland Mr Sam Adhikary, Mater Health Mr Greg McGahan, Mater Health Dr Constanze Schulz, Mater Health A/Prof Michael Beckmann, Mater Health
Name and contact information for the trial sponsor {5b}	Mater Misericordiae Limited Level 5, 41 Annerley Road Campus, South Brisbane, Qld 4101
Role of sponsor {5c}	Mater Misericordiae Limited provides resourcing and decision to submit the report for publication

## Introduction

### Background and rationale {6a}

The perinatal period is associated with a significantly increased risk of onset and relapse of mental health conditions, with self-harm being the most common cause of perinatal mortality [1, 2]. Pre- and post-natal psychiatric illness is a significant global health issue, affecting around 20% of women while remaining largely underdiagnosed and undertreated [2–5]. An estimated one in five women will experience postnatal depression or anxiety in the first year after the birth of their child [2, 6, 7]. Approximately one in 10 women will experience clinically significant symptoms of depression during pregnancy [6].

If untreated, perinatal health issues, such as depression and anxiety, can result in long-term emotional, social, and wellbeing impacts for parents, children, and families. Poor maternal mental health can lead to suboptimal infant outcomes in the future (e.g. low birth-weight infants, suboptimal development, later-life mental illness) and less favourable mother-baby bonding [8–10]. Maternal anxiety and depression during pregnancy are associated with an increased likelihood of difficult infant temperament and behavioural problems in infancy [11]. Maternal mental illness in the postpartum period has been found to impact attachment and contribute to developmental delays in motor function, language acquisition, cognitive skills, emotional self-regulation, and adaptive behaviour [12]. Early detection of psychological distress or mental illness is critical. The right treatment, care, and support can be life-changing for a mother and her infant.

Mindfulness is defined as the mental state of focusing one’s awareness on the present moment without judgement [13]. Practising mindfulness is known to have a wide range of benefits to mental wellbeing, including increased self-compassion, improved concentration, reduced rumination, and reduction in stress [14]. One intervention that appears promising in its benefits is the use of mindfulness meditation mobile applications (apps) [15]. These electronic interventions may be particularly helpful for self-managing mental health during health emergency situations, such as the recent COVID-19 pandemic, while maintaining quarantine and social distancing [16]. Online and mobile health technologies also have the potential to greatly improve access to care for patients in rural and remote areas, who experience documented disparities in maternity care and maternal health outcomes [17, 18].

Smith et al. [16] have previously examined a mindfulness intervention via a mobile app in an American prenatal population. In a randomised control trial, mothers (*n* = 80, 40 per group) who received the mindfulness app were encouraged to use it for 10 min per day for 30 days. Self-report questionnaires measuring stress (Cohen’s 10-item Perceived Stress Scale), anxiety and depression (Zigmond’s 14-item Hospital Anxiety and Depression Scale), and sleep disturbance (Patient-Reported Outcomes Measurement Information System (PROMIS) Sleep Disturbance Short Form) were administered at days 0, 14, and 30. A satisfaction questionnaire was also administered to ascertain feelings towards the mindfulness app. Participants who were in the intervention group reported lower levels of stress, anxiety and depression, and sleep disturbance following the use of the mindfulness app. Overall, satisfaction with the app was high.

While the effects of face-to-face mindfulness meditation have been studied in women in the perinatal period [15], and one study [16] has examined mindfulness via a mobile app in the prenatal population, no studies have examined the use of mindfulness app across the peripartum period. Further, no studies have explored the economic benefits of such an app. Despite this, women may be more receptive to changing their health behaviours and adopting mindfulness during pregnancy because of their concern for their unborn child [19]. The objective of this randomised controlled trial (RCT) is to assess the effect of a consumer-based mobile mindfulness app on perceived distress in maternity patients and provide an economic evaluation.

## Objectives {7}

The primary goal of the study is to assess the effectiveness of the Mater mindfulness app (a mobile app) to improve mental health outcomes in perinatal women. Secondary objectives are to evaluate the effectiveness of the app to improve mother-baby bonding, and mindful attention in users, to determine participant adherence, and to examine the economic benefits to the healthcare system of providing mindfulness support via a mobile app. The final objective will be to evaluate women’s satisfaction with the app.

## Trial design {8}

This study will be conducted in accordance with SPIRIT guidelines for clinical trial protocols. The study will be a stratified randomised controlled, superiority trial with two parallel groups and a repeated measures design. Women will be randomised into the intervention group (with full access to the Mater mindfulness app) or a passive control group (usual care). All participants will be assessed at four timepoints over the course of approximately 11 months (at 16 weeks’ gestation, 28 weeks’ gestation, 3 months postpartum, and 6 months postpartum) to assess differences between intervention and control groups. The PROMIS Global-10 survey will be used to calculate quality-adjusted life year (QALY) scores used to assess cost-effectiveness.

## Methods: participants, interventions, and outcomes

### Study setting {9}

The trial will be a single-site study conducted at the largest maternity hospital in Australia. The site, located in Brisbane, Queensland, manages an average of 10,000 births annually. This hospital provides a large range of antenatal care to women, including standard care by midwives to low-risk women, and specialty clinical care to high-risk women. A wide range of maternity models of care are available at the hospital, including specialty services for women who may be affected by substance use disorders, women with special needs, and refugee clinics. Participants for this study will be recruited from women who are attending both the public and private maternity clinics at this hospital.

### Eligibility criteria {10}

All pregnant women at less than 16 weeks’ gestation, attending the public or private maternity clinics at Mater Misericordiae in Brisbane, will be invited to participate in the study. Women must be 18 years or older and be proficient in English, as that is the only language the first iteration of the app will be created in. Women must also own a smartphone device that is capable of downloading and running the Mater mindfulness app.

### Who will take informed consent? {26a}

Eligible women will be sent a copy of the research information sheet and consent form via an SMS message. This will include a tick box where women may select to either participate or opt out of the study, and answer a question relating to the midwifery model of care, to allow for stratified randomisation. Failure to respond to the initial message will trigger a follow-up phone call by a research team member who will explain the study and request that the women select an option via the SMS-delivered consent form.

### Additional consent provisions for collection and use of participant data and biological specimens {26b}

Additional demographic and medical history data will be extracted from medical records. No sensitive or identifying data will be collected or stored on the App. De-identified survey data is collected via a university-run software program and stored on the hospital computer network.

## Interventions

### Explanation for the choice of comparators {6b}

The study compares the outcomes of an intervention group to a passive control group that undertakes usual care. A wait-list control or stepped wedge design would be inappropriate for this study as the app is tailored to specific timeframes within the pregnancy. However, all women will be given access to the app once they have completed the study.

### Intervention description {11a}

The Mater mindfulness app is a perinatal mindfulness-based app, co-designed by women with lived experience of perinatal mental illness, and clinicians with expertise in mindfulness and perinatal mental health. Final relaxation scripts were reviewed by a consumer advisory group to ensure suitability. The content of the app will consist of 40 relaxation exercises, tailored to different stages in the pre- (second trimester) and postpartum period (6 months after birth). The relaxation exercises will also be available for women to listen to on the Mater Mothers’ website.

The relaxation exercises in the app include a range of audio recordings targeting mindfulness, mother-infant bonding, relaxation, and meditation. There are also some videos of relaxation exercises to support focus. For example, the app will guide women to complete deep breathing exercises with their baby. There are also relaxation exercises completed prior to bedtime, while eating, with a partner, when pregnant, and towards the unborn baby, as well as when completing walks with and without a baby in a pram. Mindfulness-based approaches are used in a number of exercises, such as breathing in and out as women trace their fingers. See the Additional file 2 and Additional file 3 for relaxation exercise descriptions and selected transcripts. Each of the relaxation exercises is on average 3 min in length and ranging between 2 and 10 min. Each day there is one relaxation exercise that is highlighted on the home screen. On the second page of the app, there is a library of the relaxation exercises available.

The app also contains information (e.g. website, phone number, description) of a range of local and national support services that are pertinent to perinatal mental health, parenting support, child development, mental health hotlines, and partner/father mental health. The app has a mood-tracking function so that after the completion of each relaxation exercise, women rate their well-being with emoticons. Women are also affirmed with a message of encouragement once a relaxation exercise has been completed. This is recorded and viewed in a graph to assess change in mood over time. The colour scheme of the app can also be personalised based on the preferences of the woman (e.g. pink and blue, gradient from grey to green, orange and green, and turquoise and blue). The app tiles contain icons, and there is a brief description of the relaxation exercise, including the length of time for the exercise. The app is freely available and can be downloaded from the Apple and Android App Store.

### Criteria for discontinuing or modifying allocated interventions {11b}

Participants will be free to withdraw from the study at any time. Women who appear in distress will be flagged by the research team, removed from the study, and offered additional support. To ensure the validity of results, all data collected from participants up to the point of withdrawal will be included in the analysis, which will be conducted according to the intention-to-treat principle.

### Strategies to improve adherence to interventions {11c}

In order to improve adherence to the intervention requirements and to increase the completion of assessments, all surveys and study information will be sent directly to participants’ smartphone devices. Participants will receive weekly SMS messages with reminders and links to the podcasts included on the app. Surveys will be sent to participants via SMS at each of the assessment timepoints. Participants will be sent reminder messages if they do not complete the surveys within a set time period.

### Relevant concomitant care permitted or prohibited during the trial {11d}

Participants will continue to receive their routine medical care throughout the study period, regardless of being randomised to the intervention or control arm.

### Provisions for post-trial care {30}

The data collected in this study is not expected to cause significant distress or discomfort to participants. However, in the event that participants experience distress in the course of this study, the participant information sheet will provide participants with information about support services they can contact. This will include Beyond Blue, Lifeline, and the research team. In addition, participants who score highly on the EPDS scale, or noted on this scale that they have thought of self-harm, will be flagged by the research team, and further support will be offered.

### Outcomes {12}

**Fig. 1:**
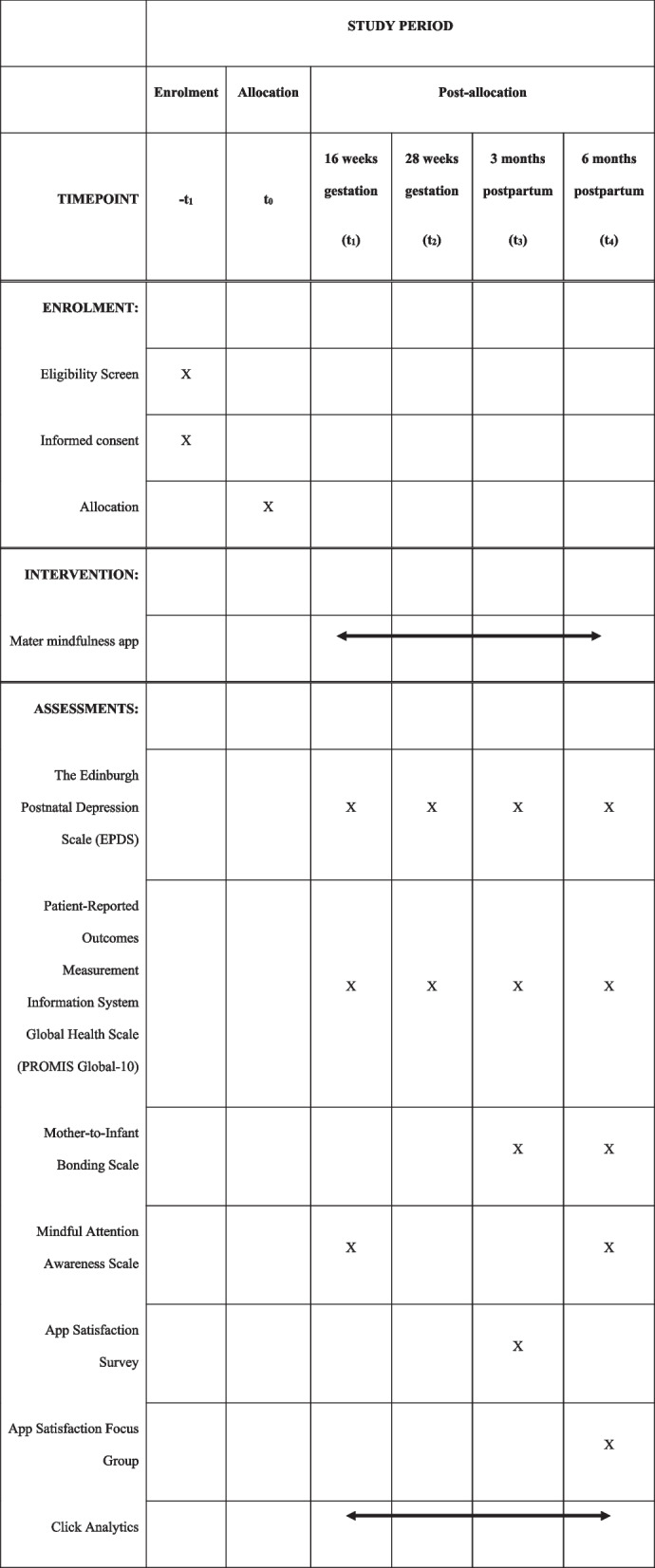
Schedule of enrolment, interventions, and assessments according to the SPIRIT 2013 guidelines

Postnatal depression will be the primary outcome of the study. This will be measured using the Edinburgh Postnatal Depression Scale (EPDS). The primary outcome will be EPDS measured at 6 months postpartum. Changes in postnatal depression over time will also be assessed and will be determined by conducting the EPDS at four timepoints: 16 weeks’ gestation, 28 weeks’ gestation, 3 months postpartum, and 6 months postpartum. Secondary outcomes measured at the same timepoints will include measures of mother-infant bonding (using the *Mother-to-Infant Bonding Scale*), quality of life, and cost-effectiveness (using the *PROMIS Global-10*). The primary timepoints for these assessments will be timepoint 1 (16 weeks’ gestation) and timepoint 4 (6 months postpartum). Mindful attention (using the *Mindful Attention Awareness Scale*) will be measured at times one and four. Participant satisfaction with the app (using a bespoke survey described below) will be measured at the third timepoint (3 months postpartum), and participant adherence (measured with click analytics to detect the frequency of app use) will be examined once participants have completed the study (see Fig. 1). The study will also use click analytics to determine the frequency of app use. Demographic data and medical health information will be extracted from medical records for the purposes of inferential statistical analyses. Changes from timepoint 1 will be assessed for all health-related, repeated surveys. Measures will be analysed in aggregate form.

### Participant timeline {13}

See the participant timeline in Fig. 1.

### Sample size {14}

The necessary sample size was calculated using the public access software G*Power 3.1.9.7 for Windows [20]. The primary outcome of depression, as measured by the EPDS, was used to estimate the required sample size. Previous meta-analyses that found mindfulness in the perinatal period reduced depression scores with an effect size of *d* = 0.59 [21, 22]. The sample size calculation was therefore based on the estimation of a medium intervention effect (*d* = 0.50). A sample size of 128 was required to compare differences at timepoint 4, with 80% statistical power, a two-tailed significance level of 5%, and a 1:1 allocation ratio. Previous studies have reported high attrition rates of 20–45% [23, 24]. Accounting for an attrition rate of 40%, a final sample size of 214, with 107 individuals in each group, will be required.

### Recruitment {15}

The study will recruit women for up to 3 months, or until the sample size is achieved. The study site manages the treatment of over 800 women per month. Given the sample requirement is less than half this amount, we anticipate achieving full recruitment within 2 months.

## Assignment of interventions: allocation

### Sequence generation {16a}

Participants will be randomly allocated to intervention or control using computer randomisation software, with a 1:1 allocation ratio. A permuted block randomisation technique (block size = 10) will be conducted to ensure assignment of participants will be even across the two groups. Randomisation will be stratified by the midwifery model of care.

### Concealment mechanism {16b}

Allocation will be concealed until assignment occurs. Consenting participants will be assigned an identification number associated with their midwifery model of care for stratified randomisation, on entry into the study. This number will be associated with a pre-defined allocation group determined by computer-generated permuted block randomisation. Recruitment and randomisation will be conducted by separate members of the research team. As such, the investigator randomising the participants will not be aware of previous or forthcoming participant allocation.

### Implementation {16c}

Randomisation will be conducted by an investigator who is not involved in recruitment or data collection. Participant information and consent SMS messages will be sent via the survey software. Upon participant enrollment and allocation, the randomisation number will be given to the investigator overseeing the distribution of the intervention.

## Assignment of interventions: blinding

### Who will be blinded {17a}

Care providers will not be aware of the assignment status of participants. Trial participants will not be blinded to their group allocation status. A member of the research team, not involved with patient care, recruitment, or group allocation, will email the intervention group instructions for downloading the online app and will provide technical assistance to participants. The data analyst will be blinded to participant allocation.

### Procedure for unblinding if needed {17b}

All participant responses will be identified with a numerical pseudonym. A coding key will be kept separate from responses in a password-protected file on the private hospital computer network. Should it become necessary, this coding key can be accessed to identify participants.

## Data collection and management

### Plans for assessment and collection of outcomes {18a}

Data will be collected through Red Cap surveys, via focus group interviews, from online medical databases, or via the app. All surveys (EPDS, PROMIS Global 10, the Mother-to-infant Bonding scale, the Mindful Attention Awareness scale, and the satisfaction survey) will be created in Red Cap survey software. Links to these surveys will be automatically sent to, and completed by, participants via SMS sent directly to their phones. The focus group interviews will collect further data relating to participant satisfaction. The online App will only collect data relating to rates and frequency.

#### The Edinburgh Postnatal Depression Scale (EPDS)

The EPDS is a 10-item questionnaire designed to screen women for symptoms of depression in the postnatal period [25]. Women completing the EPDS are asked to rate the option that best describes how they have felt in the past 7 days in response to a number of statements (e.g. “I have blamed myself unnecessarily when things went wrong.”). Items are scored on a 4-point scale, with response scores ranging from 0 to 3. Item scores are summed to produce a total score. Scores ranging from 0 to 9 indicate low levels of distress, scores from 10 to 12 indicate the presence of distress, and scores above 13 suggest a high presence of depressive symptoms. A minimum 4-point change in scores across timepoints is necessary to be deemed clinically significant [26]. The EPDS has been validated for use in both a prenatal and postnatal population [25, 27]. Women who score one, two, or three on item 10 (“The thought of harming myself has occurred to me”) will be addressed with further enquiry and support if necessary [28].

#### Patient-Reported Outcomes Measurement Information System Global Health Scale (PROMIS Global-10)

The PROMIS Global-10 is a 10-item questionnaire measuring the quality of life through a range of domains, including mental health, physical health, satisfaction with social relationships, and engagement with daily activities [29]. The first nine questions on the scale are rated on a 5-point Likert scale. Questions one to six are rated from “poor” (1) to “excellent” (5); question seven is rated from “not at all” (1) to “completely”; question eight is rated from “always” (1) to “never” (5); and question nine is rated from “very severe” (1) to “very mild” (5). Question 10 is rated on a 10-point scale, asking participants to rate pain on a scale of 1 (“no pain”) to 10 (“worst pain imaginable”). When scoring, scores on question 10 are converted onto a 5-point scale using the table provided in the scoring manual. Participants’ scores on this scale are summed to produce a raw total score. Raw scores are converted into *t*-scores for interpretation against a standardised average score. A score above 50 indicates higher than average quality of life, while a score below 50 indicates lower than average quality of life.

#### Mother-to-infant bonding scale

The Mother-to-Infant Bonding Scale is an 8-item questionnaire, which asks participants to rate their feelings about their child, to assess the level of bonding [30]. The scale presents participants with eight adjectives (e.g. loving, resentful, disappointed). Participants score how often they feel these adjectives about their child within the first few weeks. Items are scored from 0 to 3 (0 = “not at all”, 1 = “a little”, 2 = “a lot”, 3 = “very much”). Items that indicate positive emotions about the mother’s child are reverse scored (i.e. 0 = “very much”, 3 = “not at all”). A higher total score on this scale indicates lower mother-infant bonding.

#### Mindful attention awareness scale

The Mindful Attention Awareness Scale (MAAS) is a 15-item scale designed to assess participants’ attention and awareness of what is happening around them in the present [31]. The scale asks participants to rate statements about their mindful awareness (e.g. “I find it difficult to stay focussed on what’s happening in the present” and “I do jobs or tasks automatically, without being aware of what I'm doing”) on a 6-point Likert Scale from almost always to almost never. Higher average scores indicate higher mindful awareness. This scale will be used to determine whether watching weekly mindfulness-based podcasts will have a beneficial impact on mindful awareness.

#### App satisfaction

A study-specific, satisfaction survey has been developed for the purpose of evaluating satisfaction with the Mater mindfulness app. This survey is a 12-item questionnaire co-designed by clinicians and consumers, to assess women’s perceptions and opinions of the mindfulness intervention. Questions such as “how helpful did you find the Mater mindfulness app” and “the Mater mindfulness app improved my bonding with my baby” will be rated on 5- or 6-point Likert scales. This survey will be given to women in the intervention group.

A randomly selected portion of intervention participants (*n* = 10) will be invited to attend a focus group interview after the study. Semi-structured focus group interviews will be conducted in English, with no interpreter service available. An external facilitator who is experienced in conducting focus group interviews will be sought to facilitate these focus groups. Three primary questions will be asked during the focus group interviews, with prompt questions available to assist in guiding the interview if required:

i. What did you like about the Mater mindfulness app?
ii. What did you dislike about the Mater mindfulness app?
iii. What would you change about the Mater mindfulness app?

#### Click analytics

Click Analytics data will be obtained through the Mater mindfulness app to measure the frequency the app is used, how long the app is used for, and which podcasts are listened to most frequently. The app will also collect ‘mood’ data at each use of the app. This will be presented on the app as a 5-point scale in the format of happy to sad faces. No other data will be collected on the app.

### Plans to promote participant retention and complete follow-up {18b}

Participants will be entered into a draw to win one of ten $100 supermarket vouchers on completion of the study. Intervention participants who agree to participate in the focus group interview to give feedback on the app will receive a $50 supermarket voucher.

### Data management {19}

All data will be collected via survey software and stored in password-protected folders on the private Mater computer network. Email addresses, for the purpose of survey distribution and matching, will be the only identifying data collected. All surveys will be de-identified and stored in a password-protected, cloud-based server in Australia, only accessible by the participating hospital. Paper records will be stored onsite in locked files, within locked cupboards. Both of these data storage sites will only be accessible by the research team. No sensitive or identifying data (including email addresses) will be collected by, or stored on, the app.

All data will be kept for a minimum of 5 years, as per NHMRC guidelines, but may be stored longer in a de-identifiable format to be used as comparative data for future projects. When data is no longer required, it will be anonymously and privately destroyed in a shredder, or deleted from the network.

### Confidentiality {27}

Confidentiality will be assured with the use of numerical pseudonyms. No identifying information will be stored with any participant information or responses. All participants will be contacted by an external research assistant. Researchers with clinical roles or other positions within the hospital will not have any direct contact with potential or consenting participants.

### Plans for collection, laboratory evaluation, and storage of biological specimens for genetic or molecular analysis in this trial/future use {33}

NA—no biological or physical specimens will be collected for this research.

## Statistical methods

### Statistical methods for primary and secondary outcomes {20a}

The primary analyses will be conducted according to the intention-to-treat principle. This means that participant data will be analysed based on the initial treatment assignment and not the treatment ultimately received. Descriptive statistics (mean/standard deviation, median/range, proportion/percentage) will be used to describe participants’ characteristics. Normality testing by visual assessment of the data will precede inferential statistical tests of continuous outcomes, such as questionnaire scores. The primary analysis of EPDS scores at 6 months postpartum will be conducted using a linear regression model to compare the intervention and control groups. This will be analysed using an independent samples *t*-test and further sensitivity analyses using analysis of covariance (ANCOVA) to determine the impact of the intervention on EPDS scores while controlling for covariates such as age and parity.

The mean change in EPDS from timepoint 1 to timepoint 4 will also be investigated. To estimate the changes within and between test groups at each timepoint, a linear mixed effects model for repeated measures will be used. The participant will be treated as a random effect, by means of the restricted maximum likelihood method (REML). The main effects of group, time, and a group-time interaction will be analysed. To determine the effect of the baby’s sex (which will only become known at timepoint two) on outcomes, the main effects of knowledge, sex, and a knowledge-sex interaction will also be measured. Additional potential covariates (age, parity, marital status, child disability, stillbirth or infant death, and previous episode of depression) will be included as fixed effects. Secondary outcomes of interest (mother-to-infant bonding, quality of life, and mindful awareness) will be analysed with the same strategy described above.

### Interim analyses {21b}

No interim analyses are planned. Data collection will cease once the sample size has been achieved.

### Methods for additional analyses (e.g. subgroup analyses) {20b}

No additional analyses are planned. Subgroup analyses based on a stratified sample (i.e. between medical or psychiatric status or different demographic criteria) may be considered depending on the size and composition of the final sample.

### Methods in analysis to handle protocol non-adherence and any statistical methods to handle missing data {20c}

All data will be analysed using an intention-to-treat study design, regardless of protocol adherence. Missing responses within partially completed surveys will be handled as per the instructions in the scoring manual for each individual survey. Where timepoint data is missing, linear mixed model analysis will retain all data, and missing data will be assumed to be missing at random.

### Plans to give access to the full protocol, participant-level data, and statistical code {31c}

De-identifiable participant data will be stored in the University of Queensland data repository (UQRDM).

## Oversight and monitoring

### Composition of the coordinating centre and trial steering committee {5d}

The trial steering committee will consist of independent clinical members of the Catherine’s House Leadership team: the Medical Director, Nurse Unit Manager, Operational Manager, Allied Health Lead, Senior Peer Worker, Mental Health Information Manager, and Service Development and Research Team Leader. This group will meet approximately once a month.

### Composition of the data monitoring committee, its role and reporting structure {21a}

The study will designate a data monitoring committee (DMC) consisting of a minimum of four members of the Catherine’s House Leadership Team with expertise in statistical analysis, research ethics, and mental health. The DMC will meet monthly to oversee data quality, withdrawal or attrition information, and any adverse events. Access to the data will be monitored by a nominated data custodian.

### Adverse event reporting and harms {22}

All women will remain under the care of their usual treating doctors and maternity care clinicians, who will assist the women should any distress or discomfort arise. Participants will also be encouraged to inform the research team or contact one of the support service numbers listed in the information sheet, should they experience any physical or mental distress or harm for any reason while they are taking part in the research study.

### Frequency and plans for auditing trial conduct {23}

The Project Steering Committee and research team will meet every 3 months to review the progress and conduct of the trial. Annual reports will be submitted to the funding body.

### Plans for communicating important protocol amendments to relevant parties (e.g. trial participants, ethical committees) {25}

Protocol amendments will first be submitted to the Mater Human Research Ethics Committee for approval. If any changes are made that may in any way affect participants’ participation activities, involvement, or consent processes, participants will be informed of these changes by a member of the research team by email or phone.

## Dissemination plans {31a}

The authors will disseminate the results of the study at conferences and by publication in peer-reviewed journals.

## Discussion

Following the conclusion of this RCT, our results will inform the wider implementation and scalability of the app for use in other maternity services and other services across the continuum of perinatal mental health care, both nationally and internationally. This intervention can also be applied to partners and family members, as well as more vulnerable populations (e.g. women with high-risk pregnancies, increased childbirth fear, or perinatal mental illnesses). The cost-effectiveness evaluation will highlight any financial benefits of employing the app in current services.

A cost-effective or cost-saving mobile app intervention that improved mother-infant bonding and/or reduced postnatal depression would have wide-ranging benefits for healthcare services and patients. An online service would be accessible to previously inaccessible rural and remote patients or patients unable to leave the home due to health concerns or family commitments. Reductions in healthcare visits would also benefit patients in terms of the financial and time commitments necessary to travel to healthcare appointments. Reductions in postnatal depression would reduce ED presentations and therefore financial costs to the healthcare system [32]. Our cost-effectiveness evaluation will estimate these financial benefits.

## Trial status

Protocol version 3.1 (5 September 2022) was approved by the Mater Ethics Committee on 17 October 2022 (Ref: AM/MML/83589 (V3)). Recruitment for the trial will begin in November 2023 and will be completed by July 2024.

### Supplementary Information

**Additional file 1.** Participant information sheet and consent form.

**Additional file 2:** **Table 1.** Mater mindfulness app podcasts.

**Additional file 3.** Podcast example transcripts.

## Data Availability

Access to study data may be given upon request to the corresponding author.

## Abbreviations

ED: Emergency department
QALY: Quality-adjusted life years
RCT: Randomised controlled trial
