# Supplementary material for: Effectiveness and cost-effectiveness of an electronic mindfulness-based intervention to improve maternal mental health in the peripartum: study protocol for a randomised controlled trial
Source: Trials. 2023 Nov 23;24:747. doi: 10.1186/s13063-023-07746-7 (PMC10666349; doi:10.1186/s13063-023-07746-7)
Supplement: Supplementary file 1 — Additional file 1. Participant information sheet and consent form. [file 13063_2023_7746_MOESM1_ESM.docx]

**Participant Information Sheet and Consent Form**

**Mater Misericordiae Limited**

| **Title** | Effectiveness and cost-effectiveness of an electronic mindfulness-based intervention to improve maternal mental health in the peripartum: A randomised controlled trial |
| --- | --- |
| **HREC Number** | 83589 |
| **Principal Investigator** | Dr Grace Branjerdporn |
| **Location** | Mater Mothers’ Hospital, Brisbane |

1. **Introduction**

This Participant Information Sheet and Consent Form tells you about the research project. It explains the processes, tests and treatments involved. Knowing what is involved will help you decide if you want to take part in the research.

Please read this information carefully. Ask questions about anything that you don’t understand or want to know more about. Before deciding whether or not to take part, you might want to talk about it with a relative, friend or your local doctor.

Participation in this research is voluntary. If you don’t wish to take part, you don’t have to. You will receive the best possible care whether or not you take part.

If you decide you want to take part in the research project, you will be asked to sign the consent section. By signing it you are telling us that you:

• Understand what you have read

• Consent to take part in the research project

• Consent to use the intervention that is described

• Consent to the use of your personal and health information as described

You will be given a copy of this Participant Information Sheet and Consent Form to keep.

1. **What is the purpose of this research?**

The time before and after birth can be a stressful time. This study looks at the helpfulness of a mobile app (Mater Parent Lounge) that uses mindfulness to improve your mental wellbeing. We will also see if this app can promote your sleep, quality of life, and bond to your baby. This study will also see if it helps you to stay well at home, so that it reduces your need to go to the GP or hospital as often.

1. **Why have I been invited to participate in this study?**

You are invited to take part in this research because you are currently receiving care at the Mater Mothers’ Hospital antenatal clinic.

1. **Do I have to take part in this research?**

Participation in any research project is voluntary. If you do not wish to take part, you do not have to. If you decide to take part and later change your mind, you are free to withdraw from the project at any stage.

Your decision whether to take part or not to take part, or to take part and then withdraw, will not affect your routine care, your relationship with those treating you or your relationship with the Mater Mothers’ Hospital.

If you do decide to take part, you will be given this Participant Information Sheet and Consent Form to sign and you will be given a copy to keep.

1. **What does participation in this research involve?**

If you agree to participate in this study, you will be will be randomly assigned to one of two study groups: usual care or the intervention group. If you are assigned to the intervention group, you will be asked to use the Mater Parent Lounge mobile app to practice mindfulness during your pregnancy and after birth. All participants will be asked to complete questionnaires each month as part of standard care within the clinic. We will be asking questions about your level of positive and negative feelings towards your baby. We will match this to a depression and anxiety scale which you already complete as part of your routine care. We will also be collecting demographic information from your medical records, to help us understand the economic value of this mobile app. If you are randomised to the control group, you will be able to access to the app once your participation in the study has ended, for your future use.

1. **What is Mindfulness and how does it benefit me?**

Mindfulness is the act of being in a non-judgemental mindset, where you are focused on the present moment. Mindfulness can have many benefits to your wellbeing, including reduced stress, improved sleep, improved concentration, and increased kindness towards self.

1. **What is the Mater Parent Lounge app?**

The Parent Lounge app is a perinatal mindfulness-based app, co-designed by women with a lived experience of perinatal mental illness, and clinicians with expertise in mindfulness. The content on the app will consist of 40 mindfulness podcasts, tailored to different stages in the pre- (second trimester) and post-partum period (6 months after birth). The podcasts will also be available for women to listen to on the Mater Mothers website.

1. **How do I use the mobile app?**

You will be given a code to download the Mater Parent Lounge app to your mobile device. Don’t forget to use it – make sure to keep it on your home screen! You will be asked to use this app during your pregnancy and following birth. Each week, you will be given a new podcast. Please take the time during each week to listen to the podcast.

1. **What are the possible benefits of taking part?**

Your participation in this study will help us to understand how we can promote mental wellbeing in women during pregnancy and after birth. Your participation in this study will also assist in developing healthcare services to the highest level of care. Following participation in this study, you will be able to retain access to the Mater Parent Lounge app for continued use.

1. **What are the possible risks and disadvantages of taking part?**

We do not anticipate that this study will cause you any distress. If you experience distress during the course of this study, please let us know so that we can assist you.

1. **What will happen to my information?**

By signing the consent form, you consent to the research team collecting and using personal information about you for the research project. Any information obtained in connection with this research project that can identify you will remain confidential. Information collected will be stored in password protected files on the Mater server. Your information will only be used for the purpose of this research study and it will only be disclosed with your permission, except as required by law.

Information about you may be obtained from your health records held at Mater for the purpose of this research. By signing the consent form, you agree to the study team accessing health records if they are relevant to your participation in this research project.

If you withdraw from the study, we will not collect any more information about you. We would like to keep the information we have already collected about you to help us ensure that the results of the research project can be measured properly. Please let us know if you do not wish for any information to be kept.

1. **How will the results of the study be distributed?**

It is anticipated that the results of this research project will be published and/or presented in a variety of forums. In any publication and/or presentation, information will be provided in such a way that you cannot be identified, except with your expressed permission.

You can indicate on the consent form if you wish to receive a lay summary of the study findings.

1. **Who has reviewed the research project?**

All research in Australia involving humans is reviewed by an independent group of people called a Human Research Ethics Committee (HREC). The ethical aspects of this research project have been reviewed and approved by the Mater Misericordiae Ltd HREC. This project will be carried out according to the National Statement on Ethical Conduct in Human Research (2007). This statement has been developed to protect the interests of people who agree to participate in human research studies.

1. **Further information and who to contact**

The person you may need to contact will depend on the nature of your query.

If you want any further information concerning this project, you can contact the following person:

**Research contact person**

| Name | Dr Grace Branjerdporn |
| --- | --- |
| Position | Project Lead/Principal Investigator |
| Telephone | 0408 141 234 |
| Email | Grace.branjerdporn@mater.org.au |

**Mater Parent Lou App Technical Support**

If you require technical support for the Mater Parent Lounge app, please contact parentlounge@mater.org.au

If you are experiencing distress during the course of this study, we encourage you to contact the following support services:

| Lifeline | 13 11 14 |
| --- | --- |
| Beyond Blue | 1300 224 636 |
| 1300 MH CALL | 1300 462 255 |
| Research Team | 0408 141 234  [Grace.branjerdporn@mater.org.au](mailto:Grace.branjerdporn@mater.org.au) |

For matters relating to research at the site at which you are participating, the details of the local site complaints person are:

**Reviewing HREC approving this research** **and HREC Liaison Officer details**

This study has been reviewed and approved by the Mater Misericordiae Ltd Human Research Ethics Committee (EC00332). Should you wish to discuss the study in relation to your rights as a participant, or should you wish to make an independent complaint, you may contact the Liaison Officer or Chairperson, Human Research Ethics Committee, Mater Misericordiae Ltd Level 2 Aubigny Place, Raymond Terrace South Brisbane 4101 or telephone (07) 3163 1585, email: research.ethics@mater.uq.edu.au
