# Supplementary material for: Effectiveness and cost-effectiveness of an electronic mindfulness-based intervention to improve maternal mental health in the peripartum: study protocol for a randomised controlled trial
Source: Trials. 2023 Nov 23;24:747. doi: 10.1186/s13063-023-07746-7 (PMC10666349; doi:10.1186/s13063-023-07746-7)
Supplement: Supplementary file 2 — Additional file 2: Table 1. Mater mindfulness app podcasts. [file 13063_2023_7746_MOESM2_ESM.docx]

Table 1: Mater Mindfulness App Podcasts

| Podcast name | Summary |
| --- | --- |
| Basics of mindfulness | Mindfulness has many calming benefits.  Picture a box while breathing in... holding...exhaling. And holding for each side of the box. |
| Imagine Meeting Baby | Your baby is growing steadily and soon you will meet your baby face-to-face.  Take a moment to imagine what it will be like when you meet your baby. |
| Mindful Holding | Breathe deeply as you hold your baby.  Focus on your senses.  How your baby look?  What sounds does baby make?  How does it feel holding your baby? |
| Appreciating Baby | Breathe deeply as you hold your baby.  Appreciate your baby's body features. |
| Bonding with Partner | While sitting with your partner, take time to connect with each other and your baby.  Breathe deeply as you and your partner touch your baby bump. |
| Grab a Cuppa | Boiling the kettle to make a cuppa is an opportunity to practice mindfulness.  Slow down and focus on what you can see and hear as you put the kettle on. |
| Connect with the Baby | Take a moment to imagine how your baby is feeling.  Rest your hand on your baby bump and focus on your breath. |
| Tuning in to Baby | Bonding with baby begins before birth.  Sit comfortably and take a few moments to connect with your baby.  Consider your feelings towards baby and how baby is feeling. |
| 4-4-6 Breathing | Gently place your hands on your bump.  Notice the rising and falling as you breathe deeply.  Breathe in for 4...  Hold for 4...  Exhale for 6... |
| Pause for a Moment | Take a moment to pause.  Sit comfortably and breathe deeply.  Notice sensations such as what you can feel. |
| Leaves on a Stream | Take a few moments to imagine leaves gently floating down a stream.  As you do, picture putting your thoughts on each leaf and watching it float down the stream.  Let each thought float by without judgement. |
| 5 Senses | Focus on what you can see, hear, taste and touch to help ground you in the present moment.  We will start counting down from 5 things we can see. |
| Body Scan | Take some deep breaths as you bring your awareness to each part of your body.  Start with focusing on your face and gradually move down your body. |
| Hand Breathing | As you trace your fingers, let this guide your breath.  Breathe in going up your finger...  Breathe out going down your finger. |
| Eating Mindfully | Eating can be a mindful experience when we take time to notice how the food looks, tastes and feels.  Try this relax trax while eating a snack such as a sultana. |
