# Supplementary material for: Effectiveness and cost-effectiveness of an electronic mindfulness-based intervention to improve maternal mental health in the peripartum: study protocol for a randomised controlled trial
Source: Trials. 2023 Nov 23;24:747. doi: 10.1186/s13063-023-07746-7 (PMC10666349; doi:10.1186/s13063-023-07746-7)
Supplement: Supplementary file 3 — Additional file 3. Podcast example transcripts. [file 13063_2023_7746_MOESM3_ESM.docx]

Additional File 3 - Podcast Example Transcripts

**Whole Body Tension**

This relax track is about tensing and releasing your whole body.

Sit in a comfortable position.

~~~Tense everything in your whole body, stay with that tension.

Hold it as long as you can without feeling pain.

Slowly release the tension and very gradually feel it leave your body.~~~

We will now repeat this three times.

**Let’s S.T.O.P**

This relax track uses the S-T-O-P, “STOP” technique to help you when you’re feeling anxious or overwhelmed.

Let’s begin with the letter S, Stop.

Give your mind the command to stop whatever you are thinking or doing that is causing you distress.

The letter T stands for “Take a breath’.

Take slow deep breaths.

In through your nose and out through your mouth…

We will breathe in for four counts and out for six counts.

In…2…3…4…

Out…2…3….4…5…6

In…2…3…4…

Out…2…3….4…5…6

Continue reconnecting with your breath as we move to the letter O, which stands for Observe.

Observe what is happening. What is happening around you, and inside you in this moment?

What thoughts and emotions do you notice?

Be curious and open to what you observe.

For example: Say to yourself “I notice feeling angry” or “I notice the thought that I can never get on top of things”

Notice your thoughts, feelings and body sensations without judgement. Just be aware of them.

Finally, we reach P, Proceed.

Think about what you would like to do or how you would like to respond.

What’s one thing you can focus on right now?

What’s your most important and urgent priority?

Narrow down your focus and take it one small step at a time.

**SKIN TO SKIN CONTACT WITH BABY**

Skin to Skin contact with your new baby is beneficial for both of you. It involves having your baby lay on their tummy, directly on your chest, with no clothing in between.

This track relaxes your mind so you and your baby can spend time learning and connecting with each other.

Find a comfortable position preferably in a quiet and dimmed room. You may like lean against some pillows, with your shoulders relaxed, before placing your baby on your chest.

Cuddle baby close to the left side of your chest so baby can hear your heartbeat. This is good for bonding with newborns, because it helps them feel safe.

As you lay your baby down, take a deep breath in…. and a long breath out.

Watch as with each breath your chest rises and falls with your baby following along.

By using skin to skin contact, you are sharing your body temperature, bonding and talking with your baby.

If you begin to think about other things, observe these thoughts, and bring your mind back to you and your baby’s features.

Continue to breathe in, and out

In

And out.

Can you see your baby’s eyes? Maybe they are open. What colour are they? Or perhaps they are shut as your baby drifts into sleep.

Can you feel your baby’s weight? Their small body cuddling up to you. Do you feel warm?

Count each of your baby's fingers. Notice how small they are. Can you see their little nails?

If your mind wanders, bring your attention back to your baby. Keep noticing all of the small features of your baby as you continue to take deep breaths in and out.

In

And out

When you are ready, take a few moments to notice how you are feeling.

**Baby Massage**

Massage can be soothing for babies. Make sure the room is warm, your baby is quiet, well-rested and alert, and you’re relaxed.

As you do so, focus on baby and the sensations.

Smooth a few drops of baby massage oil or sorbolene cream into your warm hands.

We will start by massaging the soles of baby’s feet. Use firm, gentle, slow strokes from heel to toe.

Now baby’s legs. Do long smooth strokes up baby’s leg. Massage from ankle up to thigh and over hip. Massage both legs at once or one at a time.

Now we will massage baby’s chest. Start with massaging your baby’s shoulders. Make gentle strokes in towards the chest.

We will move to baby’s arms now. Massage baby’s arms by stroking from shoulders down towards wrists.

See if baby’s tummy feel soft. If baby’s tummy feels soft, massage baby’s tummy with circular, clockwise strokes.

Use your finger pads to massage baby’s face.

Stroke from the middle of baby’s forehead, down the outside of their face and in towards their cheeks.

Massage baby’s head in small circles.

If baby is still relaxed when you’ve finished massaging the front of their body, you can turn baby onto their tummy and use long, smooth strokes from head to toe.

Use a soothing touch.

How does baby feel?

How do you feel?
